# A new species of *Validifemur* Ma, Song & Zhu, 2007 (Lithobiomorpha, Lithobiidae) from northwest China

**DOI:** 10.3897/BDJ.10.e81849

**Published:** 2022-04-18

**Authors:** Yuhang Han, Sujian Pei, Huiqin Ma, Yaojun Li, Yinfeng Liu

**Affiliations:** 1 Institute of Myriapodology, School of Life Sciences, Hengshui University, Hengshui, China Institute of Myriapodology, School of Life Sciences, Hengshui University Hengshui China; 2 Hebei Key Laboratory of Wetland Ecology and Conservation, Hengshui, China Hebei Key Laboratory of Wetland Ecology and Conservation Hengshui China

**Keywords:** Lithobiomorpha, *Validifemur*, new species, Ningxia Hui Autonomous Region, northwest China

## Abstract

**Background:**

The myriapod fauna of China is still poorly known and very little attention has been paid to the study of Lithobiomorpha, with only more 100 species/subspecies hitherto known from the country, amongst which are only three species of *Validifemur*. Here we are describing a new species from northwest China.

**New information:**

A new lithobiid species, *Validifemurradispinipes* **sp. n.**, is described and illustrated from Wolong Mountain Park, Jingyuan County, Guyuan City, Ningxia Hui Autonomous Region, northwest China. The new species is compared with *V.pedodontus* Ma, Song & Zhu, 2007 from Shaanxi Province, China. Type specimens are deposited in the Institute of Myriapodology, School of Life Sciences, Hengshui University, Hengshui, China.

## Introduction

*Validifemur* was originally proposed as a genus of Lithobiidae in the Lithobiomorpha by Ma et al. and it accommodates only three species, all distributed in open and mixed coniferous-broadleaved forests, 550 m a.s.l., from northwest China (Ma et al. 2007a, Ma et al. 2007b; Zapparoli and Edgecombe 2011). *Validifemur* is characterised by antennae with ca. 20 articles or thereabouts; ocelli generally 1+5-1+8; forcipular coxosternal teeth 2+2, porodonts feebly thicker; all tergites without posterior triangular projections; tarsal articulation of legs 1-13 very faint or indistinct; they have very prominent secondary sexual modifications on male legs 15; female gonopods commonly with tridentate claw and usually 2+2 spurs. Presently, about 100 species/subspecies of Lithobiomorpha hitherto known from China (Wang and Mauriès 1996, Ma et al. 2014, Bonato et al. 2016, Chang et al. 2020, Pei et al. 2021a, Pei et al. 2021b, Pei et al. 2021c). The present study deals with the description and illustration of a new species of *Validifemur* from Ningxia Hui Autonomous Region, northwest China.

## Materials and methods

Specimens were collected by hand under leaf litter or stones and preserved in 75% ethanol. Illustrations and measurements were produced using a ZEISS SteREO Discovery.V20 microscope equipped with an Abbe drawing tube and an ocular micrometer and Axiocam 512 colour. The colour description is based on specimens fixed in 75% ethanol. The body length is measured from the anterior margin of the cephalic plate to the posterior end of the postpedal tergite. The terminology of the external anatomy follows Bonato et al. (2010). The type specimens examined are deposited in the Institute of Myriapodology, School of Life Sciences, Hengshui University, Hengshui, China (HUSLSIM). The following abbreviations are used in the text and in the tables: a – anterior; C – coxa; F – femur; m – median; P – prefemur; p – posterior; S, SS – sternite, sternites; T, TT – tergite, tergites; Ti – tibia; Tr – trochanter; Ta – tarsus.

## Taxon treatments

### Validifemur radispinipes

Han, Pei, Ma, Li & Li sp. n.

AF1A90DF-9E4B-5E2B-A024-82BD58B60553

CF8D2D9F-0A70-4110-BBCF-A5A97CEBF0CA

#### Materials

- **Type status:** Holotype. **Occurrence:** recordedBy: Pei Su-jian; individualCount: 1; sex: male; lifeStage: adult; **Taxon:** scientificName: *Validifemurradispinipes*; kingdom: Animalia; phylum: Arthropoda; class: Chilopoda; order: Lithobiomorpha; family: Lithobiidae; genus: Validifemur; taxonRank: species; taxonomicStatus: species; **Location:** continent: Asia; country: China; stateProvince: Ningxia Hui Autonomous Region; county: Jingyuan; locality: Wolong Mountain Park; verbatimElevation: 1958 m; verbatimCoordinates: 35°29'32.19''N 106°21'7.84''E; decimalLatitude: 35.492274; decimalLongitude: 106.352178; georeferenceProtocol: label; **Identification:** identifiedBy: Ma Hui-qin; dateIdentified: 2021; **Record Level:** collectionCode: myriapoda; basisOfRecord: PreservedSpecimen
- **Type status:** Paratype. **Occurrence:** recordedBy: Ma Hui-qin, et al.; individualCount: 39; sex: female; lifeStage: adult; **Taxon:** scientificName: *Validifemurradispinipes*; kingdom: Animalia; phylum: Arthropoda; class: Chilopoda; order: Lithobiomorpha; family: Lithobiidae; genus: Validifemur; taxonRank: species; taxonomicStatus: species; **Location:** continent: Asia; country: China; stateProvince: Ningxia Hui Autonomous Region; county: Jingyuan; locality: Wolong Mountain Park; verbatimElevation: 1958 m; verbatimCoordinates: 35°29'32.19''N 106°21'7.84''E; decimalLatitude: 35.492274; decimalLongitude: 106.352178; georeferenceProtocol: label; **Identification:** identifiedBy: Ma Hui-qin; dateIdentified: 2021; **Record Level:** collectionCode: myriapoda; basisOfRecord: PreservedSpecimen
- **Type status:** Paratype. **Occurrence:** recordedBy: Ma Hui-qin, et al.; individualCount: 45; sex: male; lifeStage: adult

#### Description

**Figure 1.**
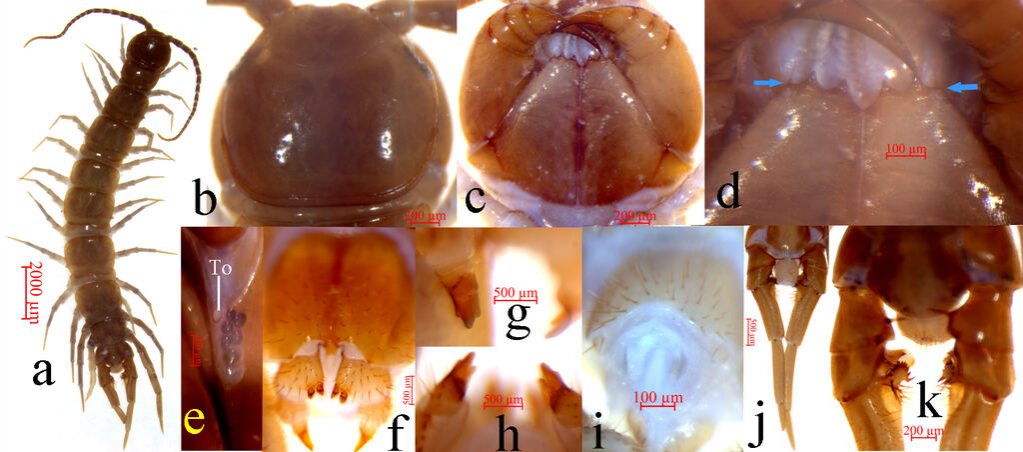
*Validifemurradispinipes* sp. n. **a** habitus, dorsal view, male holotype; **b** cephalic plate, dorsal view, male holotype; **c** forcipular coxosternite, ventral view, male holotype; **d** forcipular coxosternite, ventral view, male holotype; **e.** ocelli and Tömösváry’s organ (To), lateral view, female paratype; **f** posterior segments and gonopods in female paratype, ventral view; **g** tridentate apical claw of gonopods in female paratype, ventral view; **h** tridentate apical claw of gonopods in female paratype, dorsal view; **i** posterior segments and gonopods in male holotype, ventral view; **j** male legs 15^th^, holotype, dorsal view; **k** prefemur, femur and tibia of male legs 15^th^, holotype, dorsal view.

Body (Fig. 1a). 10.9–15.0 mm long, cephalic plate 1.3–1.5 mm long, 1.3–1.7 mm wide.

Colour. Antennae pale chestnut-brown, the pale chestnut-brown gradually becomes yellow-brown at the end of articles 4–5, terminal article yellow brown; tergites yellow-brown, cephalic plate, TT 1, 14 and 15 darker; pleural region grey; sternites pale yellow-brown; distal part of forcipules dark brown; basal and proximal parts of forcipules and forcipular coxosternite grey-brown; SS 14 and 15 yellow-brown with greyish hue; all legs pale grey-brown with pale yellowish hue; tibia more yellow, tarsus 2 pale yellow-brown on all legs.

Antennae. 20–24 articles; usually 20+20 articles, few 20+21, 20+24 articles. Length of first antennal article slightly longer than width of the base, length of the remaining articles obviously larger than wide, the distalmost articles still significantly longer than wide, 2.7–3.5 times as long as wide; abundant setae on the antennal surface, fewer on the basal articles, gradual increasing in density to approximately the sixth article, then more or less constant.

Cephalic plate. Smooth, convex, slightly wider than long; tiny setae emerging from pores scattered very sparsely over the whole surface; frontal marginal ridge with shallow anterior median furrow; short to long setae scattered along the marginal ridge of the cephalic plate; lateral marginal ridge discontinuous, posterior margin continuous, almost straight, evidently wider than lateral marginal ridge (Fig. 1b).

Ocelli. 1+6 to 1+7, commonly 1+7, oval to rounded ocelli on each side, arranged in two irregular rows, the posterior ocellus the largest. Ventral ocelli slightly smaller than the dorsal, domed, translucent and usually darkly pigmented (Fig. 1e).

Tömösváry’s organ (Fig. 1e). Close to the ocelli, situated at anterolateral margin of the cephalic plate, the surrounding sclerotised area narrow, moderately larger than the adjoining ocelli.

Coxosternite. Subtrapezoidal (Fig. 1c), anterior margin narrow, lateral margins slightly longer than medial margins; median diastema moderately deep, a slightly wider V-shape; anterior margin with 2+2 acute triangular teeth; porodonts slightly thicker, almost transparent, just posterolateral and separated from the lateral tooth, with slight bulge at base (Fig. 1c and d); long scattered setae on the ventral side of coxosternite, longer setae near the dental margin.

Tergites. Smooth, without wrinkles, dorsum slightly convex; short to long tiny setae emerging from pores scattered sparsely over the entire surface, near the margin with few long setae; TT 1 and 3 narrower than the cephalic plate, T 3 wider than the T 1. T 1 narrower postero-laterally than antero-laterally, generally inverted trapezoidal; lateral marginal ridges of all tergites continuous, TT 3–5 slightly long setae scattered sparsely over the surface. Posterior margin T1 almost straight, posterior margin of TT 3 and 5 slightly concave, posterior margin of TT 8, 10, 12 and 14 concave, posterior margin of TT 2, 4, 6, 7, 9, 11 and 13 straight (Fig. 1a). Posterior marginal ridge of TT 1 and 3 continuous, posterior marginal ridge of TT 5, 8, 10, 12 and 14 discontinuous. Posterior angles of all tergites rounded, without triangular projections. The width of TT 8 and 10 are nearly equal, but T 10 is the widest. From short to long, but miniscule setae scattered very sparsely over the surface.

Sternites. Posterior side of sternites narrower than anterior, generally inverted trapezoidal, smooth; setae emerging from sparsely scattered pores on the surface and lateral margin, very few short setae scattered sparsely amongst them; one pair of approximately symmetrically-arranged long setae on middle parts of anterior part of each sternite; with 3–5 very long setae in the anterior angles and with 1–3 very long setae in the posterior angles.

**Figure 2.**
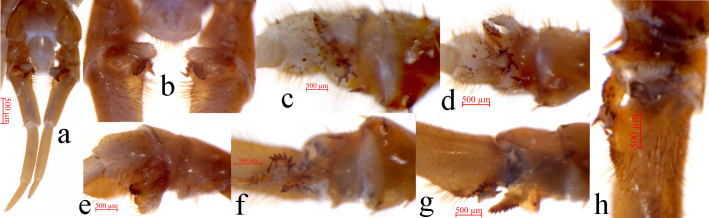
*Validifemurradispinipes* sp. n. **a** male legs 15^th^, holotype, ventral view; **b** prefemur, femur and tibia of male legs 15^th^, holotype, ventral view; **c** prefemur, femur and tibia of the right male legs 15^th^, holotype, left view; **d** prefemur, femur and tibia of the right male legs 15^th^, holotype, close to dorsal view; **e** prefemur, femur and tibia of the right male legs 15^th^, holotype, close to ventral view; **f** prefemur, femur and tibia of the left male legs 15^th^, holotype, right view; **g** prefemur, femur and tibia of the left male legs 15^th^, holotype, close to dorsal view; **h** prefemur, femur and tibia of the left male legs 15^th^, holotype, close to ventral view.

Legs. Relative robust, tarsi fused on legs 1–13, well-defined on legs 14–15. All legs with moderately long curved claws; legs 1–13 with anterior and posterior accessory spurs, anterior accessory spurs moderately long and slender, forming a moderately small angle with the claw, posterior accessory spurs slightly more robust, forming a comparatively large angle with the claw; lacking accessory spurs of legs 14 and 15, the closer to the rear of the body, the thicker the anterior accessory spurs. From short to long setae sparsely scattered over the surface of coxa, trochanter, prefemur, femur and tibia of all legs, more setae on the tarsal surfaces, especially in the ventral; setae on the dorsal and ventral surfaces slightly longer than the anterior and posterior; some thicker setae arranged in one row on the ventral surfaces of tarsi of legs 1–13, with setae significantly reduced on legs 14 and 15. Legs 14 and 15 thicker than the anterior legs in both of the female and male, male legs 15 thicker and stronger than those of the female, forming obvious secondary sexual characteristics (Fig. 1j and k; Fig. 2a and b). Ta2 3.6–5.8 times longer than wide, Ta2 65.5%–73.5% length of Ta1 on legs 15 in female; Ta2 3.9–5.4 times longer than wide, Ta2 65.1%–79.9% length of Ta1 on legs 15 in male. Leg plectrotaxy given in Table 1 and Table 2.

Coxal pores. 3–4 in a row, usually 4-4-4-3, few 3-4-4-3 in female, usually 4-4-4-1, few 4-4-5-1, 4-4-3-1, 3-4-5-4-1 or 4-4-4-2 in male; slightly oval or round, commonly round, size of coxal pore from small to large; coxal pore field set in a relatively shallow groove, the coxal pore-field fringe with a slight prominence and moderately long setae sparsely scattered over the surface.

Female. S15 anterior margin broader than posterior, generally an inverted trapezoid, postero-medially straight. Moderately long setae sparsely scattered on S15 surface. Surface of the lateral sternal margin of genital segment well chitinised, posterior margin of genital sternite deeply concave between condyles of gonopods, except for a small, median tongue-shaped bulge. Relatively long setae very sparsely scattered over ventral surface of the genital segment, slightly more setae on posterior part, especially along the posterior edge. Gonopods: first article fairly broad, bearing 18–22 moderately long setae arranged in four irregular rows, the setae on the edges are longer; with 2+2 small coniform spurs (Fig. 1f), inner spur slightly smaller than the outer; second article with 9–11 moderately long setae arranged in three irregular rows, 7–9 robust spines arranged in two irregular rows lying dorsally on the posterior part of the external margin; third article with 2–3 moderately long setae arranged in one irregular row, 3–4 robust spines arranged in two irregular rows lying dorsally on the posterior part of the external margin, with a tridentate apical claw, the largest in the middle, both sides of the dorsal and ventral sides are small and blunt. (Fig. 1g and h).

Male. S 15 posterior margin narrower than anterior, postero-medially straight, generally an inverted trapezoid, sparsely covered with long setae, the setae on the edges are longer, with a shallow nearly heart-shaped depression on the surface; sternite of genital segment evidently smaller than the female, usually sclerotised; posterior margin deeply concave between the gonopods, without medial bulge. Short to long setae equally scattered on the ventral surface of the genital segment. Gonopods short and wide, flat, with 1–2 long setae, apically slightly sclerotised (Fig. 1i). Legs 15 prominently shorter and thicker than the anterior legs, especially the coxa, trochanter, prefemur, femur and tibia; the prefemur and femur shorter and thicker, the middle dorsal spine of prefemur is almost vertical to the longitudinal axis of the body; in ventral view of femur, the anterior has a developed vesicular protuberance, the anterior surface of the protuberance is full of long bristles and the posterior of the protuberance is raised, the terminal is highly sclerotised, forming a rough spine pointing to the posterolateral side. Between of the prefemur and the femur protuberance, the femur with a finger-like protuberance and the ventral of the protuberance with 12 spurs arranged in two rows. The anterior of the tibia with a protuberance, there is an obvious depression behind the protuberance, the terminal of the protuberance is flat and has 6 radial spines; there is a longitudinal groove in the centre of the dorsal side of the tibia. The setae on the inner of the longitudinal groove are significantly thicker and longer and the density is significantly increased, the ventral of the anterior of the tibia is significantly depressed and the end of the depression is an obviously bulge and the surface is almost uniformly covered with thicker long setae (Fig. 2c-h).

Habitat. The specimens here studied were collected under the deciduous leaves of locust trees around the mountain road.

## Discussion

The new species is morphologically close to *V.pedodontus* Ma, Song & Zhu, 2007 from Shaanxi Province, SW China, with which it shares 2+2 prosternal teeth, distal claw of the female gonopods tridentate, legs 15 in male prominently thicker than the anterior legs. However, the new species can be distinguished from *V.pedodontus* easily by the following characters: 12 spurs on the femur and 6 spines on the tibia in male legs 15 vs.just11 thorns on the tibia in male legs 15 of *V.pedodontus*. T 10 is the widest of all tergites vs. TT 8 and 10 are the same length and are the widest of all tergites in *V.pedodontus*; having 7–9 robust spines arranged in two irregular rows lying dorsally on the second article of female gonopods vs. just 1 robust spines lying dorsally on the second article of female gonopods in *V.pedodontus*.

The new species can be distinguished from *V.zapparolii* Ma, Song & Zhu, 2007 from Shaanxi Province, China easily by the following characters: the femur with 12 spurs arranged in two rows, the tibia having 6 spines arranged radially in male legs 15 vs. the tibia having 10 rostriform thorns, three of them separated individually, the other thorns arranged into 2 rows in *V.zapparolii*. T 10 is the widest of all tergites vs. TT 8 and 10 are the same length and are the widest of all tergites in *V.zapparolii*; having 7–9 robust spines lying dorsally on the second article of female gonopods vs. having 3 robust spines arranged in one irregular row on the second article in *V.zapparolii*.

The new species can be distinguished from *V.digitatus* Ma, Song & Zhu, 2007 from Henan Province, China easily by the following characters: the femur with 12 spurs arranged in two rows, the tibia having 6 spines arranged radially in male legs 15 vs. the tibia having 10 sclerotised rostriform spines, forming a rough semicircle, encircling the femur in *V.digitatus*; the ocelli arranged in two irregular rows other than arranged in three irregular rows in *V.digitatus*.

## Supplementary Material

XML Treatment for Validifemur radispinipes

## Figures and Tables

**Table 1.** Leg plectrotaxy of *V.radispinipes* sp. n. (female).

Legs	Ventral					Dorsal				
	C	Tr	P	F	Ti	C	Tr	P	F	Ti
1			mp	amp	am			mp	ap	a
2-11			mp	amp	am			mp	ap	ap
12-15		m	amp	amp	am	a		amp	p	p

**Table 2.** Leg plectrotaxy of *V.radispinipes* sp. n. (male)

Legs	Ventral					Dorsal				
	C	Tr	P	F	Ti	C	Tr	P	F	Ti
1			(m)p	amp	am			mp	ap	a
2-11			mp	amp	am			mp	ap	ap
12			amp	amp	am			amp	p	ap
13-15		m	amp	amp	am	a		amp	p	p

Letters in brackets indicate variable spines.

## References

[B7655059] Bonato Lucio, Edgecombe Gregory, Lewis John, Minelli Alessandro, Pereira Luis, Shelley Rowland, Zapparoli Marzio (2010). A common terminology for the external anatomy of centipedes (Chilopoda). ZooKeys.

[B7655085] Bonato L, Chagas J A, dgecombe G D, Lewis J G E, Minelli A, Pereira L A, Shelley R M, Stoev P, Zapparoli M ChiloBase 2.0 - A world catalogue of centipedes (Chilopoda). https://chilobase.biologia.unipd.it.

[B7655268] Chang Xiao Dong, Pei Su Jian, Zhu Chun Ying, Ma Hui Qin (2020). An unusual new centipede subgenus Lithobius (Sinuispineus), with two new species from China (Lithobiomorpha, Lithobiidae). ZooKeys.

[B7655035] Ma Hui Qin, Song Da Xiang, Zhu Ming Sheng (2007). A new species of the genus *Validifemur* Ma, Song & Zhu, 2007 (Chilopoda: Lithobiomorpha) from China. Arthropoda Selecta.

[B7655009] Ma HUI QIN, Song DA XIANG, Zhu MING SHENG (2007). A new genus and two new species of lithobiid Centipedes (Chilopoda: Lithobiomorpha) from China. Zootaxa.

[B7654974] Ma HUI QIN, Pei SU JIAN, Hou XIAO JIE, Zhu TIE GANG, Wu DA YONG, Gai YONG HUA (2014). An annotated checklist of Lithobiomorpha of China. Zootaxa.

[B7655241] Pei Su Jian, Liu Hai Peng, Liang Kui Jing, Ma Hui Qin, Lu Yan Min (2021). Lithobius (Monotarsobius) tetrasulcus sp. n., a new species of centipede from China (Lithobiomorpha, Lithobiidae). Biodiversity Data Journal.

[B7655277] Pei Su Jian, Ma Hui Qin, Liu Hai Peng, Lu Yan Min, Jing Liang Kui (2021). Lithobius (Monotarsobius) femoratus sp.n., a new centipede species from China (Chilopoda: Lithobiomorpha: Lithobiidae). Arthropoda Selecta.

[B7655253] Pei Su Jian, Ma Hui Qin, Lu Yan Min, Liu Hai Peng, Liang Kui Jing (2021). A new species of *Hessebius* Verhoeff, 1941 (Lithobiomorpha, Lithobiidae) from China with a key to species. Biodiversity Data Journal.

[B7654965] Wang D Q, Mauriès J P (1996). Review and perspective of study on myriapodology of China. In: Geoffroy JJ, Mauries JP, Nguyen Duy-Jacquemin M (Eds). Acta Myriapodologica, Mémoires du Museum National d’Histoire Naturelle.

[B7655183] Zapparoli M, Edgecombe G, Minelli A (2011). Treatise on zoology–anatomy, taxonomy, biology – the Myriapoda, Volume I.

